# *Origanum syriacum* Essential Oil Chemical Polymorphism According to Soil Type

**DOI:** 10.3390/foods8030090

**Published:** 2019-03-05

**Authors:** Imad El-Alam, Raviella Zgheib, Marcello Iriti, Marc El Beyrouthy, Paul Hattouny, Anthony Verdin, Joël Fontaine, Ramez Chahine, Anissa Lounès-Hadj Sahraoui, Hassane Makhlouf

**Affiliations:** 1 Unité de Chimie Environnementale et Interactions sur le Vivant (UCEIV), Université du Littoral Côte d’Opale, SFR Condorcet FR CNRS 3417, F-62228 Calais CEDEX, France; imad.alam@hotmail.com (I.E.-A.); verdin@uni-littoral.fr (A.V.); joel.fontaine@uni-littoral.fr (J.F.); lounes@univ-littoral.fr (A.L.-H.S.); 2 Oxidative Stress and Antioxidants Group, Doctoral School of Sciences and Technologies, Lebanese University, Hadath B.P. 1500, Lebanon; charamez@hotmail.com (R.C.); drhassanemakhlouf@yahoo.fr (H.M.); 3 Institut Jean-Pierre Bourgin, Agro Paris Tech, INRA, Université Paris-Saclay, RD 10, Route de Saint-Cyr, 78026 Versailles, France; raviella-zgheib@hotmail.com; 4 Department of Agricultural and Environmental Sciences, Milan State University, via G. Celoria 2, 20133 Milan, Italy; 5 Department of Agricultural Sciences, Holy Spirit University of Kaslik, Kaslik, Jounieh B.P. 446, Lebanon; paul.elhattouny@hotmail.com; 6 Faculté de Santé Publique, Université La Sagesse, Furn-El-Chebak, Beyrouth B.P. N° 50-501, Lebanon; 7 Laboratoire Géoressources, Géosciences et Environnement—Equipe Sedre: Sol, Eau, Déchets et Ressources, Faculté des Sciences, Université Libanaise, Fanar B.P. 1200, Lebanon

**Keywords:** *Origanum syriacum*, essential oil, soil type, chemical polymorphism, arbuscular mycorrhizal fungi

## Abstract

Background: *Origanum syriacum* L. is an aromatic plant growing wild in Lebanon. This species is highly used in Lebanese traditional medicine and is a staple food in Lebanese gastronomy. Due to the over-harvesting, this species has become a cultivated crop rather than being collected from the wild. This study aims to evaluate the chemical polymorphism according to soil type. Methods: Plant samples were cultivated in different soil types including manure, potting mix, professional agriculture mixture, vegetable compost, nursery soils, and natural agricultural soil inoculated with arbuscular mycorrhizal fungi. After 16 weeks of culture, fresh shoot biomass was measured. Root colonization rate was evaluated and foliar biomasses were used for essential oil (EO) extraction. EO yield was calculated and the identification of the main chemical compounds of EO samples was performed by gas chromatography (GC) and gas chromatography–mass spectrometry (GC/MS). Results: Our findings revealed that the soil type affects the *O. syriacum* chemotype. Indeed, the EO samples could be divided into two groups: thymol chemotype group including manure and vegetable compost soils and non-sterilized non-inoculated EO samples, and the thymol/carvacrol chemotype including potting mix, professional agriculture mixture, nursery mixture, sterilized non-inoculated, non-sterilized inoculated, and sterilized inoculated EO samples. These results showed that manure and vegetable compost soils promoted thymol synthesis, whereas potting mix, professional agriculture mixture, and nursery mixture soils were thymol/carvacrol chemotype. Moreover, mycorrhizal inoculation increased carvacrol and reduced thymol productions in comparison to non-inoculated conditions. Additionally, mycorrhizal inoculation showed significant enhancements in mycorrhizal rates and shoot biomass production with respect to the non-sterilized soil. Conclusions: These variations confirm the influence of the edaphic conditions on the chemical components biosynthesis pathways of oregano plants. The results of this investigation could be used for determining optimal soil type, leading to a good quality herb production.

## 1. Introduction

Essential oils (EOs) are complex oily volatile liquids characterized by a strong odour and formed by aromatic plants as secondary metabolites. They are synthesized by different plant organs (flowers, buds, seeds, leaves, twigs, bark, herbs, wood, fruits, and roots) and are stored in secretory cells, cavities, canals, epidermic cells, or glandular trichomes [1]. EOs are widely used for bactericidal, fungicidal, insecticidal, medicinal, and cosmetic applications, especially in the pharmaceutical, cosmetic, agricultural, and food industries [2,3,4,5].

Several factors influence the quantitative and the qualitative composition of the EO: genotype, plant part, geographical location, vegetative stage, harvesting time, drying and storage conditions, extraction methods, edaphic conditions, and environmental factors, etc. [6,7,8]. These factors lead to specific chemical compositions of the EO determining EO chemotypes [9,10]. 

*Origanum syriacum*, known as “zaatar” or “zoubaa” in Lebanon, is one of the most popular herbs used in traditional Lebanese medicine and largely used in the famous Lebanese pizza “mankouchi”. It is an aromatic plant species belonging to the Lamiaceae family. Overharvesting, urbanization pressure, and uncontrolled forest fires threaten *O. syriacum* growing wild. For this reason it is now being cultivated to reduce the overexploitation of the spontaneous forms [11]. Thymol, carvacrol, p-cymene, sabinene hydrate, and γ- terpinene were identified as major constituents of *O. syriacum* EO [8,12,13], known for their antibacterial and antifungal activities, antispasmodic and radical scavenging effects, acetylcholine esterase and lipid peroxidase inhibition, white blood cell macrophage stimulation, and cardiac depressant activity [14,15,16]. Altitude, region, time of harvest, and plant part influence the composition of *O. syriacum* EO [8,12,17,18,19,20]. However, the effect of soil type on the EO chemotype is less studied. To the best of our knowledge, no articles dealing with the variation of *O. syriacum* EO chemical composition according to soil type have been published to date. Therefore, the main purpose of this work was to investigate the variability of *O. syriacum* EO chemical composition with respect to soil type. *O. syriacum* plants were cultivated in different soil types including manure, potting mix, professional agriculture mixture, vegetable compost, nursery soils, and natural agricultural soil inoculated with arbuscular mycorrhizal fungi (AMF). In fact, arbuscular mycorrhizal fungi (AMF) occur in the soil of most ecosystems. These symbiotic fungi enhance the nutritional state and water uptake of their hosts and increase disease resistance and plant health [21]. Besides, AMF may enhance EO yield and enhance the contents of some secondary metabolites of medicinal plants [22,23]. A better understanding of the effect of mycorrhizal colonization on EO chemical composition may shed light on the behaviour of plant species associated with AMF. Basic knowledge about the impact of edaphic conditions on EO chemical composition has proven to be of crucial importance for determining the optimal type of soil, leading to good quality herb production adapted to the user’s needs.

## 2. Materials and Methods

### 2.1. Experimental Design

The experiment was conducted in the greenhouse of the Faculty of Agricultural and Food Sciences of the Holy Spirit University of Kaslik (USEK) during the summer season of 2017. The greenhouse is located on the rooftop of the faculty, equipped by an automatic irrigation system that irrigates twice daily and by a ventilation system to regulate the temperature and humidity to the optimal level (25 °C, 70%). Aerial parts of *Origanum syriacum* L. was used in this study obtained from a farmer from south Lebanon. The botanical identification of plant sample was carried out by Marc El Beyrouthy according to the New Flora of Lebanon and Syria as described by [24]. A voucher specimen was deposited in the Herbarium of the Faculty of Agricultural and Food Sciences of the Holy Spirit University of Kaslik (USEK), Lebanon, under the registry number MNIII185.

Nine soil types were assessed in the current study as follows:

- soil A is 100% manure obtained from a private company in Lebanon;
- soil B is a potting mix composed of 50% peat moss and 50% potting soil;
- soil C is a professional agriculture mixture composed of 33% peat moss, 33% manure, and 33% potting soil;
- soil D is 100% vegetable compost obtained from Holy Spirit University of Kaslik;
- soil E is composed of 100% nursery mixture;
- soil F is a natural agricultural soil, collected from the Upper Litani Basin, in Marj village, Bekaa, Lebanon (latitude: 33.766404° N, longitude: 35.876819° E). A fraction of the agricultural soil (F) was sterilized by autoclave (three times at 121 °C), while the other part was not sterilized. The experiment was conducted in the presence of 30 g of an AMF inoculum, Symbivit^®^ containing six AMF morphotypes (*Claroideoglomus etunicatum*, *Glomus microaggregatum*, *Rhizophagus intraradices*, *Claroideoglomus claroideum*, *Funneliformis mosseae*, *Funneliformis geosporum*) (150,000 fungal propagule/L), or its equivalent substrate, in both sterilized and non-sterilized conditions.

The soils were dried, sieved at 2 mm and homogenized. All the conditions were repeated 10 times using 500 g of each soil type. In each condition, a seedling of *Origanum syriacum* was cultivated.

### 2.2. Plant Biomasses and Root Colonization Rate

After 16 weeks of culture, fresh shoot biomasses were measured and only foliar biomasses were directly used for EO extraction. Moreover, roots obtained on non-sterilized inoculated (NSI), non-sterilized and non-inoculated (NSNI), sterilized and inoculated (SI), and sterilized and non-inoculated (SNI) soils were rinsed and lyophilized prior measurement of their biomasses. Some of them were used for root colonization rate analysis and the rest was stored at 4 °C. In order to determine arbuscular mycorrhizal colonization of oregano plants, the roots were coloured by Trypan blue as described in [25]. Root fragments were examined with microscopic observations (×40) to detect the presence of AMF structures: arbuscules, vesicles, and intraradical hyphae.

### 2.3. EO Extraction and Yield Evaluation

EOs were extracted from total foliar biomasses obtained from all soil types using Clevenger-type apparatus for 3 h until no more EO was recovered, in accordance with the method described in the Europe Pharmacopoeia [26]. EOs were stored at 4 °C in tightly closed glass vials until their analysis. EO yield, expressed in mL/g, was calculated by measuring the volume of oil extracted per weight of fresh plant material.

### 2.4. EO Chemical Composition

Identification of the main chemical compounds of EO samples obtained on all soil types was performed by gas chromatography (GC) and gas chromatography–mass spectrometry (GC/MS) as described by [8]. 

#### 2.4.1. GC Analyses

EO composition was determined by gas chromatography (Thermo Electron Corporation apparatus equipped with flame ionization detector) using a non-polar HP-5MS (5% phenyl methyl siloxane) capillary column (30 m length, 0.25 mm inner diameter, film thickness 0.25 μm, (Supelco, Sigma-Aldrish, Darmstadt, Germany)) or a polar fused-silica HP Innowax capillary column (polyethylene glycol, 50-m length, 0.20-mm inner diameter, film thickness 0.20 μm, (Supelco, Sigma-Aldrish)). The oven temperature followed a gradient rising from 35 to 85 °C at 5 °C/min, held isothermal at 85 °C for 20 min, then rising from 85 to 300 °C at 10 °C/min, and finally held isothermal at 300 °C for 5 min; the injector temperature was 250 °C and detector temperature 310 °C. The carrier gas was He at a rate of 0.8 mL/min. Aliquots of 1 μL of diluted samples (1/100, *v*/*v*, in n-pentane (*v*/*v*)) were injected both manually and in splitless mode. 

#### 2.4.2. GC/MS Analyses

In order to confirm the identification of the components, GC/MS analyses were performed using an Agilent 6890 gas chromatograph (Agilent, Beijing, China) coupled with an Agilent 5975 mass-selective detector and equipped with an Agilent 7683 B auto sampler (injection of 1 μL of oil for each sample). The capillary columns and the GC conditions were as described above (see Section 2.4.1); Ion Source 18 and Transfer line temperatures were 310 °C and 320 °C, respectively. The mass spectrometer was in electron ionization mode at 70 eV and the mass range was: 50–400 amu (full scan mode). 

Identification and Quantification of Essential-Oil Components and Alkanes: an n-alkane (C8–C40) mixture was analysed under the same experimental GC/MS conditions to calculate the Kovats indexes (KI). Identification of individual EO components was based on comparison of their retention indices (RI), on both polar and non-polar columns, relative to the retention times (Rt) of the series of n-alkanes (C8–C40) with those of standard compounds obtained from Sigma-Aldrich (Darmstadt, Germany) or with those of the literature as described by [27,28]. Further identification was made by comparison of their mass spectra on both columns with those listed in the commercial mass spectral libraries of the National Institute of Standards and Technology (NIST; Gaithersburg, MD, USA) and Wiley 275 computer libraries, our home-made library constructed with pure compounds, or with published mass spectra as described by [27,29], allowing a reliable confirmation of the identity of each component. Standards of some EO of known composition (such as the EO of *Rosmarinus officinalis* L. from Phytosun Aroms, Plélo, France) were also injected in similar conditions for the comparison of Rt and mass spectra.

### 2.5. Statistical Analyses

All extractions and experiments were performed in triplicates. Significant differences between the means of the shoot and root biomasses, root colonization rate, as well as EO composition were assessed according to Fisher’s least significant difference (LSD) test using Statgraphics Centurion 15.2.11.0 (Statgraphics Technologies, Warrenton, VA, USA).

Besides, the effect of inoculation and sterilization on root dry weights was tested by Student’s *t*-test using the SPSS 16.0 software (SPSS Inc., Chicago, IL, USA). Results were considered significantly different at the 0.05 level (*p* < 0.05). Moreover, the effect of soil types on EO composition was tested by the principal component analysis (PCA) using XLSTAT 2014.5.03 (Addinsoft, Barcelona, Spain).

## 3. Results

### 3.1. Plant Biomasses and Root Mycorrhizal Rates

The plant development differed with respect to soil type. Indeed, total foliar biomasses obtained on different soil types ranged between 4.2 g for sterilized-inoculated soil and 533 g for nursery mixture soils (Table 1).

On the other hand, on sterilized soil, no difference was revealed between shoot biomass of inoculated (1.15 g) and non-inoculated (0.85 g) plants, while on non-sterilized soil, shoot biomass was significantly higher for inoculated oregano plants (3.52 g) in comparison with non-inoculated ones (1.59 g) (Fisher’s least significant difference (LSD) test: *p* < 0.05) (Table 2).

On the other hand, the root biomasses obtained on non-sterilized soil were 0.83 and 0.85 g for non-inoculated and inoculated conditions, respectively. Besides, root biomasses obtained on sterilized soils were 0.65 g and 0.80 g for non-inoculated and inoculated conditions, respectively. No significant difference was observed between the root dry weight of inoculated and non-inoculated plants grown in sterilized or non-sterilized soils (Fisher’s least significant difference (LSD) test: *p* > 0.05).

Microscopic observations of stained *Origanum syriacum* root samples obtained from all soil types revealed the presence of AMF structures: arbuscules, vesicles, and intraradical hyphae. Total root mycorrhizal rates were about 51% and 23% for inoculated oregano cultivated respectively on non-sterilized and sterilized soils (Table 2). No AMF colonization was observed in the absence of inoculation on sterilized soil, while root colonization rate was about 17% on the non-inoculated and non-sterilized soils.

### 3.2. EO Extraction and Yield Evaluation

Table 1 presents EO yields of *O. syriacum* cultivated in the different soil types studied. Yields of EO extracted from oregano plant varied according to soil type and fluctuated between 0.1% and 4.1% (Table 1). The highest yield was obtained in sterilized and inoculated sample, while the lowest one was registered in 100% manure soil sample.

### 3.3. EO Chemical Composition

In total, 35 components were identified in the volatile oil of *O. syriacum* plants cultivated in 9 different types of soil and representing 90.7–98.4% of the total oil composition. Table 3 gathers the composition of the EO of the nine samples of oregano collected. The compounds were grouped into five classes: monoterpene hydrocarbons, oxygenated monoterpenes, sesquiterpenes hydrocarbons, oxygenated sesquiterpenes, and others. Monoterpenes, both monoterpene hydrocarbons, and oxygenated monoterpenes were the most highly represented classes. The main components were: αp-cymene (4.2–14.8%), γ-terpinene (1.5–10.6%), thymol (37.8–56.3%), carvacrol (10.3–35.8%), and β-caryophyllene (1.2–2.1%) (Table 3). Our results showed that *O. syriacum* EO obtained from all soil types were distinguished by the levels of thymol and carvacrol. Indeed, high levels of thymol were identified in manure and vegetable compost soils (soils A and D), as well as non-sterilized and non-inoculated (NSNI) EO samples, whereas the EO samples obtained on nursery mixture soil (soil E), potting mix and professional agriculture mixture (soils B and C), sterilized-non inoculated (SNI), non-sterilized inoculated (NSI), and sterilized inoculated (SI) soils contained high levels of thymol and carvacrol.

**Figure 1:**
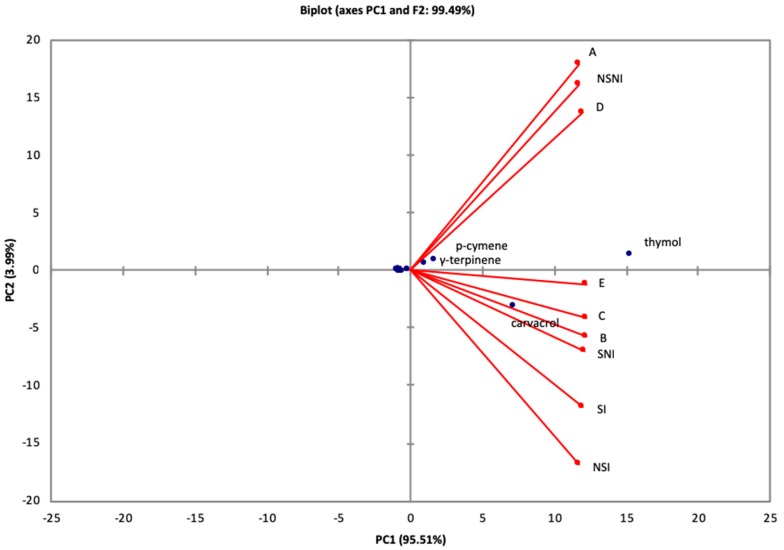
Principal component projection plot of principal components PC1 and PC2 scores and loadings indicating chemotypes within *Origanum syriacum* based on chemical composition of the essential oils.

In addition, the principal components analysis (PCA), a multivariate statistical method, was conducted, using the relative proportion of *O. syriacum* EO constituents, in order to (1) extract more valuable information, (2) investigate the separation between samples and the main chemical constituents, (3) evaluate the chemical variability according to the type of soil, inoculation, and sterilization and (4) classify the chemotype. Figure 1 represents the main results as obtained by PCA revealing that the first two principal components accounted for 99.49% of the variability.

The EO samples were clearly separated into two groups. A separation of the EO samples is clearly shown along principal component 2 (PC2). EO extracted from oregano cultivated in 100% manure and vegetable compost soils, as well as non-sterilized and non-inoculated soils were distributed in a group with relatively higher proportion of thymol as a principal constituent marking thymol chemotype. The EO samples obtained from nursery mixture soil, potting mix and professional agriculture mixture, sterilized-non inoculated soil, non-sterilized-inoculated soil, and sterilized-inoculated soil were characterized by the chemotype: thymol/carvacrol. PCA highlighted the existence of two different chemotypes in *O. syriacum* EO study samples, proving the existence of chemical variability: thymol and thymol/carvacrol.

## 4. Discussion

*Origanum syriacum* EO composition is known to be affected by altitude, region, and time of harvest, as well as part of the plant [8]. The current study aims to evaluate the effect of soil type on *O. syriacum* EO chemotype.

Our results showed that the yield of extracted EO ranged from 0.1% to 4.1% in all tested soil types. These findings are in agreement with those found in the literature [30,31,32]. This observed variability in the yield production of the EO extracted from *O. syriacum* cultivated in different types of soil could be related to the interaction between edaphic factors, nutrient availability and mycorrhizal inoculation. Indeed, organic matter, water balance, and mineral rate (P and N) are known to promote EO yields of medicinal plants [31,33,34,35]. In particular, the use of biofertilizers such as compost and manure enhanced aromatic plant growth and EO productivity [36,37,38]. Besides, AMF symbiosis enhanced EO yield by promoting plant growth as well as nutrient and water uptake [22,23,39].

On the other hand, the analysis of the main compounds of oregano EO signalled the composition variability within soil types. Indeed, with regard to their chemical composition, the obtained EO could be divided into two groups: (1) the thymol chemotype group including manure and vegetable compost soils and non-sterilized non-inoculated EO samples, and (2) the thymol/carvacrol chemotype including potting mix and professional agriculture mixture, nursery mixture soil, sterilized non-inoculated EO samples, non-sterilized-inoculated samples, and sterilized-inoculated EO samples.

In the same way, our findings showed that the manure and vegetable compost soils promoted thymol synthesis, whereas potting mix, professional agriculture mixture, and nursery mixture soils were of the thymol/carvacrol chemotype. These variations confirm the influence of the edaphic conditions on the chemical components’ biochemical pathways of oregano plants. Indeed, EO yield and terpenoids synthesis can be influenced by nutrient availability in the soil [40,41,42]. The authors of [43] proved the effect of N and P treatments on the main constituents of *Lavandula angustifolia* (Mill.) essential oil (1.8-cineole, borneol, camphor, α-terpineol, myrtenal). The increase in the amount of aluminium, copper, iron, potassium, manganese, phosphorus, and sulphur in soil affected the amount of EO of *Thymus pulegioides* and the biosynthesis of the main compounds [44]. The authors of [45] found a positive correlation coefficient between N and essential oil contents in Egyptian oregano (*Origanum syriacum* L. var. *aegyptiacum* Tackh). Nitrogen fertilization seemed to increase the biosynthesis of thymol and carvacrol. However, contrasting results were obtained indicating that organic fertilizers such as compost and manure may present significant influence on the EO chemical composition in medicinal plants [36,38,46,47].

Moreover, our results highlighted the effect of mycorrhizal inoculation on EO composition and showed that the concentration of carvacrol was higher in inoculated conditions with respect to non-inoculated ones, while the concentrations of thymol decreased with inoculation. Therefore, inoculation may orient biosynthesis towards the preferential formation of a specific constituent leading to a defined chemotype by activating specific enzymes or group of enzymes. Indeed, AMF symbiosis is known to enhance the contents of some secondary metabolites of medicinal plants [39] by increasing the density of glandular trichomes [48] and enhancing the phosphorus plant nutrition [23,39]. Nevertheless, there are contrasting investigations indicating that AMF symbiosis did not have any significant influence on the EO chemical composition in medicinal plants [22,23,39]. Besides, after 16 weeks of culture, moderate spontaneous AMF oregano root colonization rates were observed in the agricultural soil (non-sterilized and non-inoculated soil). This is due to the presence of autochthonous AMF spores in the soil. Based on morphological characters, three AMF species were identified in the studied agricultural soil (*Funnelliformis mosseae*, *Septoglomus constrictum* and *Claroideoglomus lamellosum*). Addition of commercial mycorrhizal inoculum in the soil led to significant improvement of colonization rate of *O. syriacum* roots in comparison with non-inoculated conditions. Nevertheless, in spite of the moderate colonization rate, arbuscular mycorrhizal inoculation significantly increased the shoot biomass of oregano plants but only in non-sterilized soil. It is well know that arbuscular mycorrhizal fungi improve plant growth by (1) exploring a larger volume of soil, (2) enhancing the mineral (phosphate, nitrogen, oligo-elements) and water nutritional state of their hosts, and (3) alleviating biotic and abiotic stresses such as pollution in host plants [49,50]. Besides, the presence of both AMF, as well as soil microorganisms such as plant growth-promoting rhizobacteria (PGPR) strains in non-sterilized soil may explain this observation [51,52].

The results of this investigation could be used to select the appropriate soil type for the optimal production of *O. syriacum* EO and meet the needs of the users.

## 5. Conclusions

Results presented herein revealed that chemical composition of *Origanum syriacum* EO could be affected by soil type as well as by the presence of mycorrhizal inoculation. Indeed, manure and vegetable compost soils increased thymol synthesis, whereas potting mix, professional agriculture mixture, and nursery mixture soils were of the thymol/carvacrol chemotype. Besides, the AMF symbiosis promoted carvacrol synthesis and decreased the concentration of thymol. Therefore, this study contributes to present data for *O. syriacum* cultivation, establishes parameters to essential oil components, and responds to the needs of the users in order to provide a high economic return. Further assays should be conducted in order to improve the quantity and quality of the EO obtained on tested soil types, by searching for appropriate AMF species, oregano variety, and soil type composition. 

## Figures and Tables

**Table 1:** Total foliar biomass measurements and essential oil yields of *Origanum syriacum* harvested from different soil types.

Soil Type	A	B	C	D	E	F			
						NSNI	NSI	SNI	SI
Total foliar biomass fresh weight (g/pot)	31.4	303.4	115.4	8.1	553.4	11.4	20.1	4.2	4.9
Essential oil volume (mL)	0.3	1.8	0.8	0.15	4.9	0.4	0.3	0.1	0.2
Yield (mL/g)	0.1	0.6	0.7	1.9	0.9	3.5	1.5	2.4	4.1

Soil A: 100% manure; soil B: potting mix composed of 50% peat moss and 50% of potting soil; soil C: professional agriculture mixture composed of 33% peat moss, 33% manure and 33% potting soil; soil D: 100% vegetable compost; soil E: 100% nursery mixture; soil F: natural agricultural soil; NSNI: non sterilized-non inoculated soil; NSI: non sterilized-inoculated soil; SNI: sterilized-non inoculated soil; SI: sterilized-inoculated soil.

**Table 2:** Plant biomasses and root mycorrhizal rates obtained in agricultural soil (soil F).

	NSNI	NSI	SNI	SI
Shoot dry weight (g/plant)	1.59 ± 1.56 ^a^	3.52 ± 1.89 ^b^	0.85 ± 0.37 ^a^	1.15 ± 0.85 ^a^
Root dry weight (g/plant)	0.83 ± 0.82 ^a^	0.85 ± 0.46 ^a^	0.65 ± 0.28 ^a^	0.80 ± 0.59 ^a^
Mycorrhizal rate (%)	17.03 ± 3.3 ^a^	51.1 ± 4.7 ^b^	0 ^a^	22.9 ± 4.6 ^a^

Values are depicted as mean ± standard deviation. Different letters indicate significant differences at *p* < 0.05 as determined by Fisher’s least significant difference (LSD) test.

**Table 3:** Chemical composition of *Origanum syriacum* essential oil.

Soil type	A	B	C	D	E	F			
EO composition (%)						NSNI	NSI	SNI	SI
α-pinene	nd	nd	nd	nd	nd	1.47 ± 0.03 ^a^	1.56 ± 0.03 ^a^	0.77 ± 0.06 ^ab^	nd
camphene	nd	nd	0.1	nd	nd	0.24 ± 0 ^a^	0.17 ± 0.002 ^a^	nd	nd
sabinene	nd	nd	nd	nd	nd	0.32 ± 0.01 ^a^	0.30 ± 0.06 ^a^	0.20 ± 0 ^c^	0.13 ± 0 ^d^
β-pinene	nd	0.5	0.2	nd	nd	nd	nd	nd	nd
1-octen-3-ol	nd	nd	0.4	nd	0.4	0.20 ± 0.003 ^a^	0.30 ± 0.10 ^ab^	0.35 ± 0.04 ^b^	0.35 ± 0.04 ^b^
myrcene	nd	1.3	1.4	nd	1.7	0.87 ± 0.002 ^a^	1.64 ± 0.38 ^b^	0.32 ± 0 ^c^	0.32 ± 0 ^c^
3-octanol	nd	nd	nd	nd	nd	0.17 ± 0.002 ^a^	nd	0.23 ± 0 ^c^	0.23 ± 0 ^c^
α-phellandrene	nd	nd	nd	nd	nd	0.43 ± 0.005 ^a^	0.70 ± 0.26 ^b^	0.24 ± 0 ^a^	0.24 ± 0 ^a^
**α-terpinene**	**2.5**	**1.5**	**2.7**	**2.4**	**2.5**	**2.10 ± 0.03 ^a^**	**2.27 ± 0.46 ^a^**	**1.13 ± 0.06 ^b^**	**0.76 ± 0 ^b^**
**p-cymene**	**12.6**	**4.9**	**5.6**	**9.4**	**4.2**	**14.83 ± 0.47 ^a^**	**7.50 ± 1.35 ^b^**	**5.97 ± 0.06 ^c^**	**4.47 ± 0.12 ^d^**
β-phellandrene	nd	0.7	0.4	nd	1.2	nd	nd	nd	nd
trans-β-ocimene	nd	nd	0.2	nd	nd	nd	0.2 ± 0^a^	nd	nd
**γ-terpinene**	**11.4**	**6.5**	**4.7**	**10.6**	**6.4**	**3.83 ± 0.08 ^a^**	**6.04 ± 0.77 ^b^**	**1.56 ± 0.03 ^c^**	**1.56 ± 0.03 ^c^**
cis-sabinene hydrate	nd	0.6	nd	nd	nd	0.68 ± 0.06 ^ab^	0.55 ± 0.37 ^a^	0.80 ± 0 ^ab^	0.98 ± 0.10 ^a^
terpinolene	nd	nd	0.3	nd	nd	0.11 ± 0.01 ^ab^	0.15 ± 0.04 ^b^	0.09 ± 0 ^a^	0.09 ± 0 ^a^
α-terpineol	nd	nd	nd	nd	nd	0.26 ± 0.02 ^a^	0.08 ± 0.04 ^b^	0.10 ± 0 ^b^	0.10 ± 0 ^b^
borneol	nd	nd	0.1	nd	nd	nd	nd	nd	nd
neo-allo-ocimene	nd	nd	nd	nd	nd	nd	nd	nd	nd
terpinen-4-ol	nd	nd	nd	nd	nd	nd	0.16 ± 0.10	nd	nd
thymol methyl ether	nd	nd	0.2	nd	nd	nd	nd	nd	nd
thymoquinone	nd	0.5	1.2	nd	nd	0.81 ± 0.07 ^a^	0.29 ± 0 ^b^	1.20 ± 0 ^c^	1.70 ± 0 ^d^
**thymol**	**54.3**	**46.5**	**47.8**	**56.3**	**51.2**	**54.48 ± 0.03 ^a^**	**37.80 ± 1.56 ^b^**	**48.03 ± 0.12 ^c^**	**43.83 ± 0.42 ^d^**
**carvacrol**	**10.3**	**28.5**	**27.4**	**14.1**	**25.9**	**11.46 ± 0.17 ^a^**	**35.84 ± 1.68 ^b^**	**30.13 ± 0.06 ^c^**	**32.47 ± 0.71 ^c^**
carvacryl acetate	nd	nd	0.2	nd	nd	nd	nd	nd	nd
**β-caryophyllene**	**2.1**	**1.3**	**2.5**	**3.1**	**1.2**	**2.14 ± 0 ^a^**	**1.93 ± 0.12 ^b^**	**1.73 ± 0.06 ^ac^**	**1.85 ± 0.05 ^c^**
α-bergamotene	nd	nd	nd	nd	nd	nd	0.08 ± 0.01	nd	nd
α-humulene	nd	0.7	0.6	nd	nd	0.19 ± 0 ^a^	0.34 ± 0.02 ^b^	0.06 ± 0 ^c^	0.06 ± 0 ^c^
germacrene D	nd	nd	0.1	nd	nd	0.07 ± 0 ^a^	0.09 ± 0.01 ^b^	0.10 ± 0 ^b^	0.30 ± 0 ^c^
γ-cadinene	nd	0.6	0.1	nd	nd	nd	0.08 ± 0^b^	0.10 ± 0 ^c^	0.10 ± 0 ^c^
δ-cadinene	nd	nd	nd	nd	nd	nd	0.06 ± 0^b^	0.50 ± 0 ^c^	0.90 ± 0 ^d^
delta-cadinene	nd	nd	nd	nd	nd	nd	nd	nd	nd
cis-alpha-bisabolene	nd	0.3	0.2	nd	nd	nd	nd	nd	nd
caryophyllene oxide	nd	0.7	0.9	nd	nd	0.19 ± 0 ^a^	0.10 ± 0 ^b^	0.20 ± 0 ^c^	0.30 ± 0 ^d^
humulene-1,2-epoxide	nd	0.4	0.1	nd	nd	nd	nd	nd	nd
α-cadinol	nd	nd	0.5	nd	nd	nd	0.17± 0.13	nd	nd
**Total**	**93.2**	**95.5**	**97.9**	**95.9**	**94.7**	**94.85**	**98.4**	**93.8**	**90.7**

nd: not detected. Values are depicted as mean ± standard deviation. Different letters indicate significant differences at *p* < 0.05 as determined by Fisher’s test of EO chemical composition obtained on agricultural soil (soil F).
